# Highland Barley Tartary Buckwheat Coarse Grain Biscuits Ameliorated High-Fat Diet-Induced Hyperlipidaemia in Mice Through Gut Microbiota Modulation and Enhanced Short-Chain Fatty Acid Secretion Mice

**DOI:** 10.3390/foods14122079

**Published:** 2025-06-12

**Authors:** Xiuqing Yang, Xiongfei Kang, Linfang Li, Shaoyu Zhang

**Affiliations:** 1 Institute of Biotechnology, Shanxi University, Taiyuan 030006, China; 2 College of Life Sciences, Shanxi University, Taiyuan 030006, China; 202223110008@email.sxu.edu.cn (X.K.); 202223110015@email.sxu.edu.cn (L.L.); 202223110043@email.sxu.edu.cn (S.Z.)

**Keywords:** highland barley tartary buckwheat coarse-grain biscuits, dietary intervention, lipid metabolic, gut microorganisms, reducing blood fat

## Abstract

Dietary modification plays a crucial role in managing and preventing hyperlipidemia. This study examined the combination of highland barley, tartary buckwheat, mung beans, Ormosia hosiei, black rice, and corn germ oil in multi-grain biscuit form. This formulation leverages the synergistic interactions among bioactive compounds, which exert preventive and therapeutic effects against lipid disorders. C57BL/6N mice were fed a high-fat diet for 12 weeks to establish a hyperlipidemia model, followed by feeding with highland barley tartary buckwheat coarse-grain biscuits for 4 weeks. The experimental outcomes revealed that the highland barley tartary buckwheat coarse-grain biscuits effectively controlled body weight and reduced fasting blood sugar levels: body weight was restored to approximately 29 g, and the fasting blood sugar level returned to the normal range of 6 mmol/L. We also observed improved organ indices and regulated blood lipids in hyperlipidemic mice. The total cholesterol of high-fat mice was reduced to 5 mmol/L and the triglyceride level to 1 mmol/L. A significant reduction in inflammatory markers and histopathological improvement in hepatic and adipose tissues were also observed. The intervention enhanced leptin and adiponectin secretion while elevating concentrations of acetic, propionic, butyric, valeric, and caproic acids. Microbiome analysis demonstrated favorable shifts in bacterial populations, characterized by increased Bacteroidetes and Verrucomicrobia abundance and a decreased Firmicutes-to-Proteobacteria ratio, promoting beneficial genera while suppressing potentially pathogenic taxa. These findings suggest that the developed highland barley tartary buckwheat coarse-grain biscuits are a promising dietary intervention for hyperlipidemia management. The effects were potentially mediated through gut microbiota modulation and enhanced short-chain fatty acid production. This research provides novel insights into functional food development for hyperlipidemia.

## 1. Introduction

Hyperlipidemia poses a major public health challenge globally. It is characterized by an abnormal increase in plasma TC, TG, and LDL-C levels, and an abnormal decrease in HDL-C levels; metabolic imbalance is aggravated, leading to lipid accumulation in the viscera and causing chronic inflammation and oxidative stress damage. Epidemiological studies indicate that its prevalence exceeds 25% across populations and that it continues to rise [1,2]. Current therapeutic approaches primarily involve dietary modifications and pharmacological interventions [3]. Among lipid-lowering medications, statins have demonstrated superior efficacy in managing dyslipidemia [4,5]. These agents function by enhancing HDL-C levels while effectively lowering TC, LDL-C, and TG, establishing them as a first-line therapeutic option [6,7]. Despite its efficacy, statin therapy has several limitations, including the composition of a substantial economic burden and documented adverse effects [8].

In contrast to pharmacological interventions, dietary approaches based on everyday nutrition have gained greater public acceptance for managing health conditions [9]. Scientific investigations have highlighted natural food sources as particularly promising for addressing hyperlipidemia. Scientists worldwide have reported numerous bioactive compounds in the human diet capable of reducing blood lipid disorders [10]. Evidence indicates that dietary modifications can substantially decrease the prevalence of hyperlipidemia while mitigating disease progression. This approach offers advantages over prolonged pharmaceutical use, which may involve adverse effects [11]. Emerging evidence further proposes that enhancing whole-grain consumption contributes to gut microbiome diversity, suggesting dietary adjustment as a viable strategy for combating various chronic metabolic disorders [12].

Intestinal microorganisms refers to the vast number of microorganisms in the intestinal tract of animals. These microorganisms live in the intestinal tract of animals and help the host complete a variety of physiological and biochemical functions. Additionally, gut microbiota play a crucial role in the body’s fat metabolism. Gut microbes not only affect the digestive tract but can also influence the liver through the gut–liver axis. A high-fat diet may alter the structure of gut microbial communities, thereby disrupting metabolism and destabilizing fat metabolism, inhibiting the proliferation of probiotics, and leading to a significant decrease in beneficial bacteria and an increase in harmful bacteria. Short-chain fatty acids (SCFAs), also known as volatile fatty acids, are divided into straight and branched chains. SCFAs originate from beneficial microorganisms in the gut, such as lactic acid bacteria and bifidobacteria, which break down indigestible carbohydrates and dietary fiber to produce these substances. They are closely related to the host metabolism and the improvement of the intestinal barrier function, positively impacting gut health. These acids can adjust the distribution of gut microbiota, enhance immune function, and also help regulate body fat content.

Existing research on non-alcohol fatty liver disease (NAFLD) and hyperlipidemia has predominantly focused on single-grain interventions. Liu et al. found that β-glucan from highland barley exhibits therapeutic potential against hepatic steatosis in NAFLD mouse models [13]. The experimental findings of Li indicated that black-rice modulated lipid profiles mitigated hepatic injury and reduced adipose deposition in hyperlipidemic mice [14]. Nutritional studies conducted by Hou et al. revealed that Vigna radiata consumption counteracted diet-induced obesity while improving gut microbiota diversity in mice [15]. Li et al. proposed that millet’s hypolipidemic mechanism may involve enhanced SCFA production and the transcriptional regulation of lipid metabolism genes [16]. Zhou further demonstrated the anti-obesity effects of tartary buckwheat through gut microbial community restructuring in dietary obesity models [17]. These studies illustrate the therapeutic potential of whole grains. However, systematic exploration of the synergy among multiple grains is lacking.

A growing body of research has focused on the link between dyslipidemia and gut microbial imbalance. Fiber-rich cereals such as oats and barley have demonstrated the potential to modulate intestinal flora [18]. However, due to their unique texture and taste, these grains are not widely consumed on a daily basis. Blending diverse grains offers nutritional complementarity, enhances synergistic phytochemical interactions, and improves sensory acceptability [19]. Despite this, the current literature lacks substantial investigations into the combined effects of grain mixtures—such as highland barley, tartary buckwheat, mung beans, Ormosia hosiei, and black rice—in biscuit form on the gut microbiota. Notably, emerging evidence highlights nutrient synergy within natural food matrices in promoting health [20,21]. Whole grains have been shown to reduce protein fermentation in the gut microbiome and may increase microbial diversity [22,23]. Building upon these findings, our research takes a novel approach by developing multi-grain biscuits incorporating highland barley, tartary buckwheat, mung beans, Ormosia hosiei, black rice, and corn germ oil. Unlike what studies on commercial mixed-cereal products lacking mouse experiments have shown, this study systematically investigated the anti-hyperlipidemic mechanisms of these biscuits through analyzing lipid metabolism, evaluating oxidative stress, and profiling the gut microbiome in a diet-induced hyperlipidemic murine model. Specifically, we examined how these biscuits mediated lipid regulation through intestinal flora modulation and short-chain fatty acid secretion. This research explores potential mechanisms to inform dietary interventions for dyslipidemia, and the results may provide guidance for the remission of hyperlipemia.

## 2. Materials and Methods

### 2.1. Materials and Reagents

The biscuits were purchased from Lan County Quanmin Food Factory (Lanxian, China). The ingredients included highland barley, tartary buckwheat, corn oil, xylitol, Ormosia hosiei, black rice, mung beans, and baking soda. The nutritional content per 100 g was as follows: sucrose 0 g, trans fatty acids 0%, dietary fiber 6.98 g, β-glucan 2.03 g, protein 8.10 g, fat 26.0 g, carbohydrates 61 g, sodium 54 mg, energy 2137 kJ, calcium 31.9 mg (per 100 g), iron 5.92 mg (per 100 g), zinc 1.57 mg (per 100 g), and selenium 0.0024 mg (per 100 g) [24].

Regular feed with the following composition was purchased from the Institute of Radiation Protection of China(Taiyuan,China): carbohydrates 64.5%, protein 19.54%, water 2.47%, fiber 2.14%, fat 4.3%, ash 4.65%, phosphorus 1%, and calcium 1.4%. We fed the mice in the NC group with this kind of feed. High-fat feed (30% lard, 10% whole milk powder, 8% sucrose, 1% cholesterol, and 0.5% sodium cholate) was sourced from Shanghai Shuyu Biotechnology Co., Ltd. (Shanghai, China).

Lovastatin was purchased from Chengdu Yongkang Pharmaceutical Co., Ltd. (Chengdu, China). Additionally, TC, TG, LDL-C, HDL-C, ALT, and AST were purchased from Zhongqing Zhongyuan Huiji Biotechnology Co., Ltd. (Chongqing, China), while the MDA, SOD, CAT, GSH-Px, IL-6, TNF-α, ADP, and LEP for the enzyme-linked immunosorbent assays were supplied by the Shanghai Veneptide Biotechnology Co., Ltd. (Shanghai, China).

### 2.2. Animals and Experimental Design

Forty-eight male C57BL/6N mice aged 4 weeks (body mass 18 ± 2 g) were sourced from the Beijing Vitonglihua Laboratory Animal Technology Co., Ltd. (certification number SCXK (Beijing) 2021-0006, Beijing, China). The mice were housed under controlled conditions with ambient temperatures regulated between 20 and 26 °C, with humidity levels maintained at 40–70% and a standardized photoperiod cycle (12-h light/dark intervals). Through randomized allocation, six experimental cohorts were established, each consisting of eight mice. Housing arrangements included four animals per cage, with individual identification achieved through ear tagging. Weekly monitoring protocols included cage sanitation procedures, quantitative assessments of somatic growth parameters, and dietary intake measurements.

**Figure 1:**
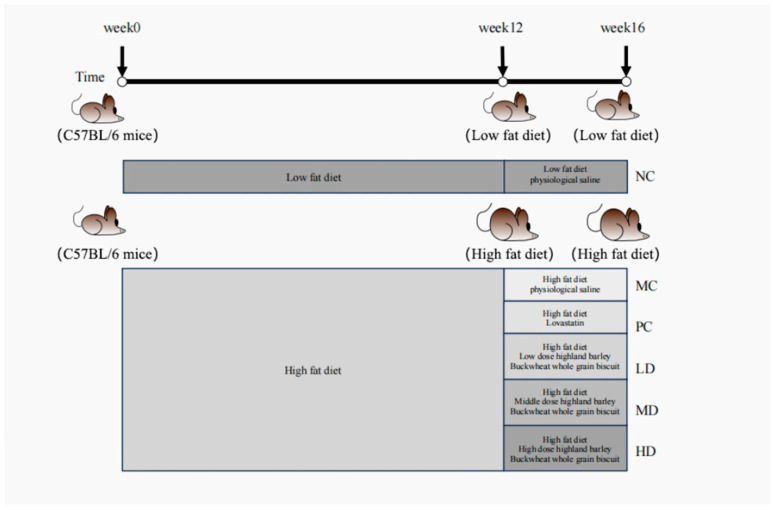
Schematic diagram of high-fat mouse model establishment and dietary intervention design.

The experimental design is shown in Figure 1. The mice were acclimatized for 7 days. After excluding those with abnormal body weight due to environmental incompatibility, 46 mice remained. These mice were randomly allocated into two groups: a normal control group (NC, n = 10) and a high-lipid model group (HL, n = 36). The NC group was maintained on a standard nutritional diet, whereas the HL group received a lipid-enriched feed to induce hyperlipidemia (Duration: 12 weeks). All mice had ad libitum access to water throughout the study.

After completing the 12-week dietary regimen, the experimental animals underwent a 12 h fasting period with continued access to water. Subsequently, their body weights were recorded, and fasting blood glucose concentrations were measured through standardized methods. Blood samples were collected via the orbital sinus for lipid panel analysis. The model validation criteria included the following: body weight in the HL group surpassing that of the NC group ≥ 20%, TC concentration at or above 5.20 mmol/L, TG levels exceeding 1.20 mmol/L, and substantially elevated fasting glucose measurements compared to the NC group. Upon meeting these parameters, four NC and six HL group mice were randomly chosen and euthanized via CO₂ asphyxiation. Histopathological evaluations on liver and epididymal adipose tissue specimens were performed, which showed marked disparities in hepatic pathology and adipocyte hypertrophy between the experimental groups, confirming successful establishment of the hyperlipidemic model [25].

The high-fat diet cohort (HL) was stratified into five experimental subgroups (n = 6/group): high-fat control (MC), positive control (PC), low-dose intervention (LD), medium-dose intervention (MD), and high-dose intervention (HD). Throughout the study period, HL mice remained on the high-fat regimen, while the NC group was fed the standard chow. Daily intervention consisted of the same amount of normal saline administration for the NC and MC groups, with the PC cohort receiving a lovastatin solution (10 mg/kg BW/day). The experimental groups (HD, MD, and LD) received daily intragastric doses of biscuits at 1.66, 1.25, and 0.83 g/kg body weight, respectively (duration: 4 weeks). All mice underwent continuous gastric gavage administration for the 28-day experimental duration.

Upon completion of the experimental protocol, all mice underwent a 12 h fasting period with ad libitum access to water. Blood glucose was determined through tail vein blood sampling using a precision glucometer. Subsequently, mice received intraperitoneal anesthesia with 4% chloral hydrate (0.1 mL/10 g body weight) prior to retro-orbital sinus blood collection. Collected specimens underwent refrigerated centrifugation (4 °C, 3000× *g*, 15 min) for serum isolation and biochemical analyses. After deep anesthesia, mice were euthanized by cervical dislocation. Visceral organs, including myocardial, hepatic, splenic, renal, pulmonary, and epididymal adipose tissues, were excised for weight measurement. Selected hepatic and epididymal fat were fixed in 4% paraformaldehyde for subsequent histological processing and microscopic evaluation. The collected samples were rapidly frozen in liquid nitrogen and stored in a −80 °C refrigerator for subsequent experimental analysis.

### 2.3. General Indicator Observations

Weekly measurements of body weight and dietary intake were conducted, while behavioral patterns and psychological states were systematically monitored through observational assessments.

### 2.4. Measurement of Organ and Fat Indices in Mice

Cardiac, hepatic, splenic, renal, pulmonary, and epididymal adipose specimens underwent saline rinsing. Following thorough cleansing, surface moisture was absorbed using filter paper prior to comprehensive tissue processing. Organ mass ratio and adipose tissue indices were subsequently determined through gravimetric analysis; refer to this literature [26].

The organ and adipose indices were determined utilizing the following formula:

Hepatic index (mg/g) = fresh liver tissue mass (mg)/terminal body mass (g)

Adipose index (mg/g) = fat mass (mg)/final body weight (g)

### 2.5. Serum Tests and Liver Homogenate Analyses

Following centrifugation, murine serum samples were collected and subjected to automated biochemical assessment using automatic biochemical equipment (Thermo Fisher Scientific, Waltham, MA, USA) to measure the TC, TG, HDL-C, LDL-C, ALT, and AST concentrations. Liver specimens (0.1 g) were transferred to a glass homogenizer (Shanghai Ansairui Industrial Co., Ltd. Shanghai, China) and mixed with a nine-fold volume of physiological saline. The homogenization process was conducted using the mechanical disruption method under ice-cooled conditions. Subsequent centrifugation parameters included 3500 revolutions per minute for 15 min at 4 °C, followed by supernatant collection. The extracted hepatic samples were then analyzed to determine the CAT, GSH-Px, SOD, and MDA concentrations.

### 2.6. Detection of Serum IL-6, TNF-α, LEP, and ADP

Mouse serum samples were harvested. IL-6, TNF-α, adipokines, and leptin concentrations were quantified through enzyme-linked immunosorbent assay (ELISA).

### 2.7. Histological Analysis

Mouse liver and epididymal adipose specimens were preserved in a 10% formalin solution, followed by paraffin embedding and microtome sectioning. The tissue sections were then subjected to hematoxylin and eosin (H&E) staining and examined using a standard light microscope (Olympus Corporation, Tokyo, Japan).

### 2.8. Analysis of Cecal Short-Chain Fatty Acid Composition

The determination of short-chain fatty acids was modified somewhat based on the method proposed by Hu et al. [27]. Calibration standards for volatile fatty acids (acetic, propionic, butyric, caproic, and valeric acid) were prepared. Then, cecal contents (0.1 g) were mixed with 500 μL deionized water and 100 mg glass beads, followed by mechanical homogenization (1 min) and centrifugation (10 min, 12,000× *g*, 4 °C). A 200 μL aliquot of supernatant subsequently underwent the stepwise addition of 100 μL of 15% H3PO4, 20 μL of internal standard (4-methylvaleric acid, 375 μg/mL), and 280 μL of diethyl ether. The mixture was then vortexed for 30 s and re-centrifuged (5 min, 12,000× *g*), after which the organic phase was collected for GC analysis. Analyses were conducted using a Thermo Trace 1310 GC system equipped with an Agilent HP-INNOWAX column (30 m × 0.25 mm × 0.25 μm) in split mode (10:1 ratio, 1 μL injection). The oven temperature program was as follows: initial 90 °C (2 min hold), ramped at 10 °C/min to 120 °C, then at 5 °C/min to 150 °C, followed by 25 °C/min to 250 °C (2 min final hold). Helium served as the carrier gas (1.0 mL/min constant flow rate) with the injector and transfer line temperatures maintained at 250 °C.

### 2.9. Analysis of the Fecal Microflora

The microbial analysis of fecal samples included sample collection, DNA extraction and amplification, 16S rDNA sequencing, construction of a PCR product library, and high-throughput sequencing.

### 2.10. Statistical Analysis

All experimental data are presented as the mean ± standard deviation (SD) based on three independent measurements. Statistical comparisons across experimental groups were conducted using SPSS software (version 17.0), with a significance threshold set to *p* < 0.05.

## 3. Results

### 3.1. Determination of Model Evaluation Index in Hyperlipidemic Mice

**Figure 2:**
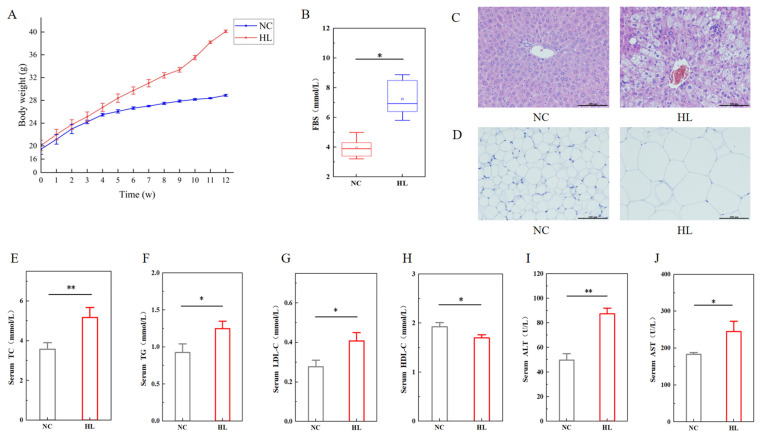
Determination of mold-making evaluation index in hyperlipidemic mice. (**A**) Effect of high-fat diet on the body weight of mice, (**B**) effect of high-fat diet on fasting blood glucose in mice, (**C**) results of H&E staining of liver sections from each group of mice (200×), (**D**) results of H&E staining of epididymal fat sections of each group of mice (200×), (**E**) influence of high-fat diet on the level of TC in serum, (**F**) influence of high-fat diet on the level of TG in serum, (**G**) influence of high-fat diet on the level of LDL-C in serum, (**H**) influence of high-fat diet on the level of HDL-C in serum, (**I**) influence of high-fat diet on the level of ALT in serum, and (**J**) influence of high-fat diet on the level of AST in serum. NC vs. HL: *p* < 0.05, *; *p* < 0.01, **.

A total of 48 male C57BL/6N mice aged 4 weeks were acquired for this study. Following 7 days of acclimatization and the elimination of outliers, 46 mice meeting the standard weight criteria were retained. Weekly body mass recordings (Figure 2A) revealed comparable baseline weights between the NC and HL cohorts, confirming appropriate randomization. After 12 weeks, the HL group exhibited a 38.75% greater average mass (40.1 g) than the NC group (28.9 g). Biochemical analyses demonstrated a significant elevation (*p* < 0.05) in serum TC, TG, LDL-C, ALT, and AST levels, alongside a reduced HDL-C concentration in the HL group mice relative to the NC group (Figure 2E–J). Fasting glucose measurements showed marked hyperglycemia in the HL cohort (Figure 2B; *p* < 0.05). Histopathological examination revealed distinct hepatic lesions and epididymal adipose tissue hypertrophy in high-fat-fed animals compared to the NC group (Figure 2C,D). These metabolic disturbances and morphological alterations confirmed successful induction of hyperlipidemia in the experimental model [28].

### 3.2. Effects of Biscuits on Weight Gain, Food Intake, Caloric Intake, and Fasting Blood Glucose in Hyperlipidemic Mice

**Figure 3:**
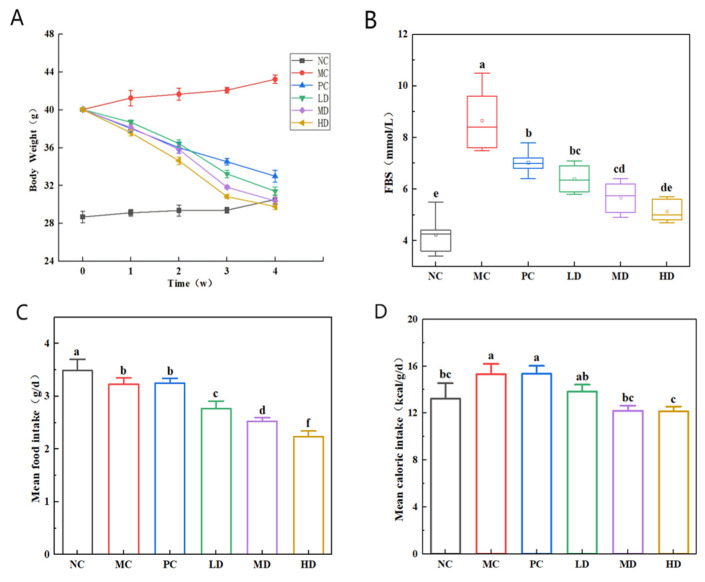
Effects of biscuits on weight gain, food intake, caloric intake, and fasting blood glucose in hyperlipidemic mice. (**A**) Effect of biscuits on body weight in hyperlipidemic mice, (**B**) effect of biscuits on fasting blood glucose in hyperlipidemic mice, (**C**) food consumption in each group, and (**D**) caloric intake in each group. NC vs. MC: the different lowercase letters at the top of the graph bars represent statistical significance (*p* < 0.05). MC vs. PC, LD, MD, and HD: the different lowercase letters at the top of graph bars represent statistical significance (*p* < 0.05).

Comparable initial body weights were observed across the high-fat diet groups, confirming accurate group randomization (Figure 3A,B). Following 4 weeks of dietary intervention, the MC group exhibited a marked elevation in body weight and fasting glucose relative to the NC control group (*p* < 0.05). The observed metabolic changes likely resulted from murine adiposity caused by the energy-dense nature of the high-fat diet coupled with the enhanced secretion of adipocyte-derived factors that induce insulin resistance and subsequent hyperglycemia. All intervention groups (LD, MD, HD, and PC) demonstrated significant suppression of weight gain and glucose levels compared to the MC group (*p* < 0.05). Notably, those in the HD group approximated those in the NC group, with no significant differences observed (*p* > 0.05). The body weight returned to a normal level of approximately 29 g, and the fasting blood sugar level returned to the normal range of 6 mmol/L. These findings demonstrate that consumption of biscuits effectively mitigated weight gain and glycemic increase in hyperlipidemic mice.

Metabolic energy expenditure drives weight changes, and when bodily caloric intake fails short of metabolic demands, adipose tissue is mobilized to meet energy needs, leading to weight loss [29]. As shown in Figure 3C,D, distinct dietary patterns emerge across groups. The NC mice exhibited the highest daily food consumption and had the lowest caloric intake, markedly exceeding the other groups in quantity but exhibiting significantly reduced energy absorption (*p* < 0.05). This was due to the consumption of standard chow in the NC group, as the diet had superior palatability and lower energy density compared to the high-fat alternatives. To satisfy their metabolic requirements, these mice consequently increased their feed consumption.

Comparative data demonstrated that the MC group animals had lower food consumption and a higher caloric intake compared to the NC group (*p* < 0.05). The PC cohort, which received lovastatin, exhibited comparable feeding patterns to the MC group (*p* > 0.05). Notably, the LD, MD, and HD experimental groups, which received biscuits, exhibited significantly diminished food consumption and caloric intake relative to the MC control group (*p* < 0.05). This phenomenon may be attributed to the elevated dietary fiber content of the biscuits, which prolongs satiety and effectively curtails feeding behavior, thereby mitigating weight gain in the hyperlipidemic mice.

### 3.3. Effects of Biscuits on Organ Index and Fat Indices in Hyperlipidemic Mice

In this study, hepatic enlargement was markedly greater in the high-fat MC group compared to the NC group (Table 1; *p* < 0.05). Intervention with varying doses of highland barley tartary buckwheat coarse-grain biscuits or lovastatin administration effectively counteracted diet-induced hepatomegaly, with the MD and HD groups exhibiting the most pronounced attenuation. This suggests that the addition of the doses of the highland barley tartary buckwheat coarse-grain biscuit had a protective effect on the liver. Renal analysis revealed a significant increase in organ weight in the MC group (*p* < 0.05). Notably, cardiac indices in the PC group showed a marked reduction compared to the other groups (*p* < 0.05), which was potentially attributable to pharmacological adverse effects. Splenic and pulmonary parameters remained unaffected across all dietary conditions. The most substantial metabolic impact was observed in the epididymal adipose, where consumption of the high-fat diet doubled adiposity indices in the MC group versus the NC group (*p* < 0.05). Therapeutic interventions in the PC, LD, MD, and HD groups significantly reduced fat accumulation relative to the MC control group (*p* < 0.05). Efficacy was comparable between the HD and PC groups. This suggests that ingestion of biscuits effectively mimics the pharmacological effect of lovastatin in this murine model.

### 3.4. Effects of Biscuits on Serum Index in Hyperlipidemic Mice

**Figure 4:**
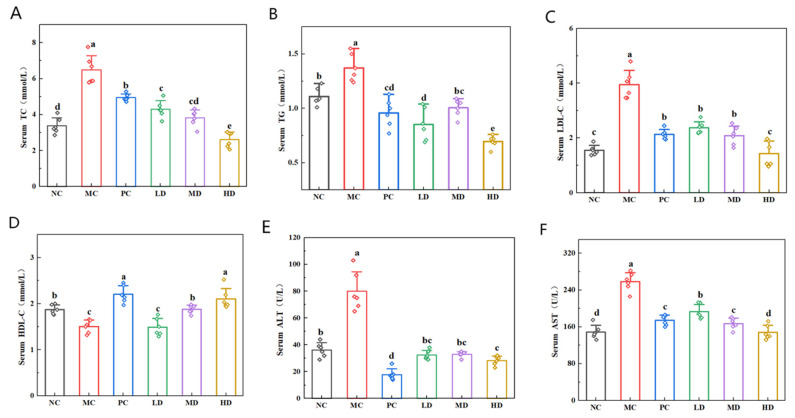
Effects of biscuits on serum indices in mice. (**A**) Influence of biscuits on the level of TC, (**B**) influence of biscuits on the level of TG, (**C**) influence of biscuits on the level of LDL-C, (**D**) influence of biscuits on the level of HDL-C, (**E**) influence of biscuits on the level of ALT, and (**F**) influence of biscuits on the level of AST. NC vs. MC: the different lowercase letters at the top of graph bars represent statistical significance (*p* < 0.05). MC vs. PC, LD, MD, and HD: the different lowercase letters at the top of graph bars represent statistical significance (*p* < 0.05).

The mice in the MC group exhibited substantially higher serum TC, TG, and LDL-C levels and significantly lower HDL-C levels than the NC group (Figure 4A–D; *p* < 0.05). Subsequent administration of highland barley tartary buckwheat coarse-grain biscuits or lovastatin therapy resulted in markedly reduced TC, TG, and LDL-C values across treatment groups relative to the MC group (*p* < 0.05). The total cholesterol of high-fat mice was reduced to 5 mmol/L and the triglyceride level to 1 mmol/L. The low-density lipoprotein cholesterol of high-fat mice was reduced to approx. 2 mmol/L. HDL-C was significantly elevated in the MD, HD, and PC groups compared to the MC group (*p* < 0.05). The high-density lipoprotein cholesterol of high-fat mice was raised to approx. 2 mmol/L. Notably, the HD group exhibited superior lipid modulation. This was demonstrated in the form of significantly lower TC, TG, and LDL-C concentrations (*p* < 0.05) and equivalent HDL-C levels compared to the PC group (*p* > 0.05). These findings substantiate the lipid-regulating efficacy of biscuits in counteracting diet-induced dyslipidemia.

ALT and AST concentrations are widely recognized as crucial biomarkers for assessing hepatic health in clinical diagnostics. Elevated serum concentrations of these enzymes can serve as a biochemical marker of hepatic injury severity. As illustrated in (Figure 4E,F), the MC group exhibited marked elevation in ALT and AST levels compared to the NC group (*p* < 0.05). This demonstrates that prolonged high-fat dietary intake induced hepatocyte damage in this murine model. Notably, the therapeutic intervention groups (PC, LD, MD, and HD) showed statistically significant reductions in ALT and AST levels relative to the MC group (*p* < 0.05). The ALT of high-fat mice was reduced to 40 U/L and the AST level to 180 U/L. This confirmed that both the lovastatin treatment and consumption of biscuits effectively mitigated diet-induced hepatic impairment. The HD and PC groups demonstrated optimal therapeutic outcomes, with ALT and AST concentrations approaching those of the NC group. These results suggest that the biscuits exerted comparable hepatoprotective efficacy to the pharmacological intervention, effectively regulating serum enzyme profiles, preventing hepatic functional deterioration, and maintaining liver homeostasis.

### 3.5. Effects of Biscuits on Oxidative Stress in the Livers of Hyperlipidemic Mice

Investigations have demonstrated that hyperlipidemic mice commonly exhibit signs of oxidative stress [30]. Extended intake of a diet rich in both lipids and carbohydrates has been shown to promote excessive generation of reactive oxygen species in mice, initiating cellular oxidative stress mechanisms that result in hepatic impairment. Evidence has also revealed reciprocal connections between lipid-rich nutritional patterns and oxidative stress processes, establishing bidirectional causality in these pathological interactions [31].

**Figure 5:**
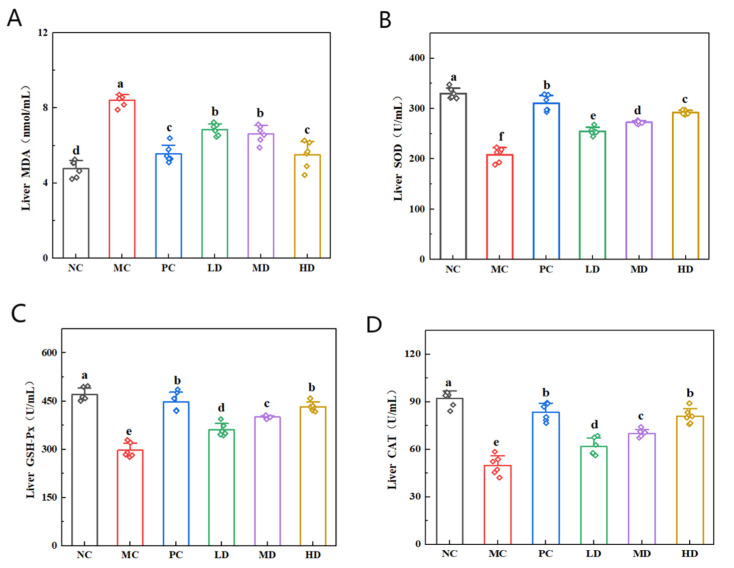
Effects of biscuits on oxidative stress in the liver of hyperlipidemic mice. (**A**) Influence of biscuits on the level of MDA in the liver, (**B**) influence of biscuits on the level of SOD in the liver, (**C**) influence of biscuits on the level of GSH-Px in the liver, and (**D**) influence of biscuits on the level of CAT in the liver. NC vs. MC: the different lowercase letters at the top of graph bars represent statistical significance (*p* < 0.05). MC vs. PC, LD, MD, and HD: the different lowercase letters at the top of graph bars represent statistical significance (*p* < 0.05).

In this study, the MC group exhibited a marked reduction in SOD, CAT, and GSH-Px relative to the NC group (Figure 5; *p* < 0.05), with MDA levels showing a significant elevation (*p* < 0.05). The intervention groups (PC, LD, MD, and HD) demonstrated a substantial increase in SOD, CAT, and GSH-Px activity compared to the MC group (*p* < 0.05) and a statistically significant reduction in MDA (*p* < 0.05). Notably, the levels of the above oxidative stress-related indicators in the HD and PC groups were close to those in the NC group. These results suggest that biscuits effectively counteracted high-fat-induced oxidative stress, strengthened endogenous antioxidant capacity, and enhanced physiological protection [32].

### 3.6. Effects of Biscuits on Inflammation-Related Factors in Hyperlipidemic Mice

**Figure 6:**
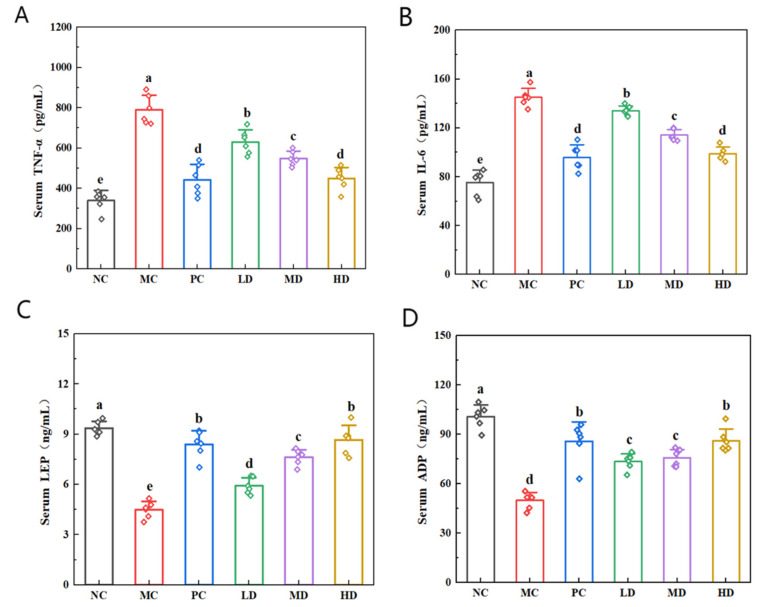
Effects of biscuits on inflammation-related factors in hyperlipidemic mice. (**A**) Influence of biscuits on the level of TNF-α, (**B**) influence of biscuits on the level of IL-6, (**C**) influence of biscuits on the level of LEP, and (**D**) influence of biscuits on the level of ADP. NC vs. MC: the different lowercase letters at the top of graph bars represent statistical significance (*p* < 0.05). MC vs. PC, LD, MD, and HD: the different lowercase letters the top of graph bars represent statistical significance (*p* < 0.05).

Consumption of a high-fat diet can induce inflammation, leading to an elevated concentration of pro-inflammatory cytokines in the bloodstream [32]. IL-6 and TNF-α serve as key mediators of cellular inflammatory processes. During inflammatory activation, intracellular production of these cytokines escalates substantially, thereby entering the systemic circulation and manifesting as a heightened serum concentration [33]. Figure 6A and B illustrate the markedly elevated IL-6 and TNF-α levels in the MC group compared to the NC group (*p* < 0.05). Therapeutic intervention with biscuits or lovastatin significantly reduced cytokine concentrations in the PC, LD, MD, and HD treatment groups relative to the MC group (*p* < 0.05).

Leptin, a hormone produced by adipose cells, plays an essential role in combating excessive weight accumulation. Adiponectin is another adipocyte-derived signaling molecule that has been shown to improve the lipid profile and contribute to vascular health. Figure 6C,D illustrate the marked reduction in ADP and LEP concentration in the MC group compared to the NC group (*p* < 0.05). Subsequent intervention demonstrated that dietary supplementation with biscuits or lovastatin administration resulted in significantly elevated ADP and LEP levels in the PC, LD, MD, and HD treatment groups compared to the untreated MC group (*p* < 0.05). Notably, the HD group exhibited comparable therapeutic efficacy to the PC group, with no statistically significant intergroup variation observed (*p* > 0.05). These findings suggest that the biscuits effectively modulated the inflammatory serum biomarkers IL-6 and TNF-α while enhancing LEP and ADP secretion. The therapeutic mechanism likely involved dual regulation of the leptin and adiponectin pathways in this hyperlipidemic murine model. These findings offer a potential nutritional intervention strategy for lipid metabolism disorders.

### 3.7. Effects of Biscuits on Histopathology in Hyperlipidemic Mice

**Figure 7:**
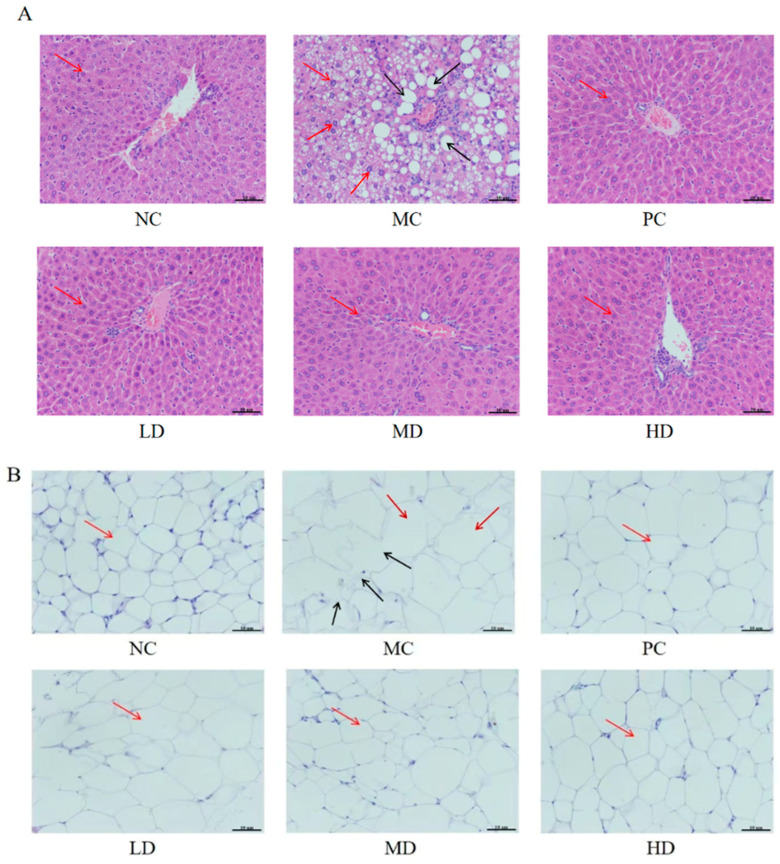
Effects of biscuits on histopathology in hyperlipidemic mice. (**A**) Results of H&E staining of liver sections from each group of mice (200×),and (**B**) results of H&E staining of epididymal fat sections of each group of mice (200×). In (**A**), the red arrow in the NC, PC, LD, MD and HD group represents the normal cell, the red arrows in the MC represent cells with accumulating fat granules, the black arrows reprensent vacuolated cells. In (**B**), the red arrow in the NC, PC, LD, MD and HD group represents the normal cell, the red arrows in the MC group represent cells with increased volume, the black arrows represent the blurred boundaries of cells.

The liver serves as the primary organ responsible for the metabolic processing of lipids and cholesterol [29]. H&E staining and histological examination of hepatic tissues in this study revealed normal cellular boundaries with distinct cellular stratification and no lipid vacuoles in the NC group. Conversely, the MC group exhibited blurred cellular boundaries, hepatocyte enlargement, cytoplasmic lipid droplet accumulation, and extensive vacuolization. This demonstrates that prolonged high-fat dietary intake triggered significant lipid metabolism disorder and hepatic steatosis. Comparative analysis showed that biscuit intervention or lovastatin administration in the PC, LD, MD, and HD groups resulted in considerable mitigation of these pathological hepatic alterations. These therapeutic groups demonstrated well-organized hepatocyte architecture, complete elimination of intracellular lipid deposits, and absence of adipocyte vacuolization (Figure 7A). These findings suggest that supplementation with the biscuits effectively counteracted high-fat diet-induced lipid deposition and ameliorated hepatic pathological changes in hyperlipidemic mice.

Histopathological findings from the murine epididymal adipose tissues are shown in (Figure 7B). H&E staining demonstrated that adipocytes in the NC group exhibited uniform size, orderly arrangement, and distinct cellular margins, with relatively compact cell morphology. In contrast, adipocytes in the MC group exhibited disorganized spatial distribution and heterogeneous volumetric expansion accompanied by indistinct peripheral demarcations. Following intervention with biscuits or lovastatin administration, the PC, LD, MD, and HD groups demonstrated progressive cellular reorganization. Adipocytes in these experimental groups exhibited normalization size, moderate cytoplasmic compaction, well-defined borders, and reduced dimensions compared to the MC group. These morphological improvements suggest that dietary incorporation of biscuits may effectively regulate pathological adipocyte hypertrophy and mitigate adipose tissue degeneration processes.

Histological observation of hepatic and epididymal adipose tissues aligned with the biochemical parameters. These data demonstrated that biscuits effectively mitigated lipid deposition in these organs while alleviating tissue damage. This pathological evidence corroborates the therapeutic potential of the biscuits in regulating lipid metabolism disorders.

### 3.8. Effects of Biscuits on Short-Chain Fatty Acids in the Cecal Contents

**Figure 8:**
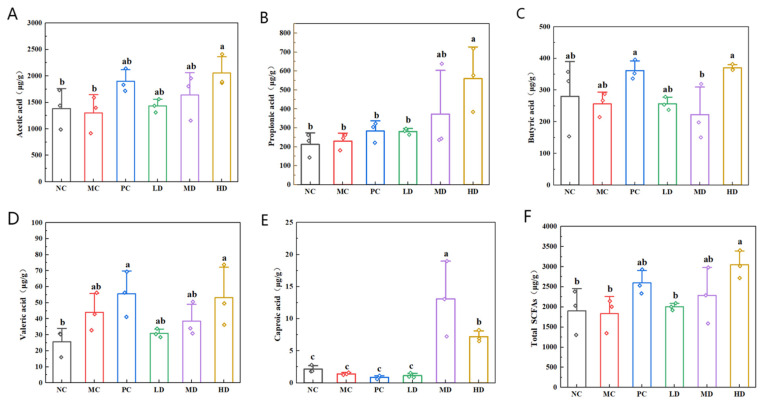
Effects of biscuits on short-chain fatty acids in mouse cecal contents. (**A**) Influence of biscuits on the level of acetic acid, (**B**) influence of biscuits on the level of propionic acid, (**C**) influence of biscuits on the level of butyric acid, (**D**) influence of biscuits on the level of valeric acid, (**E**) influence of biscuits on the level of caproic acid, and (**F**) influence of biscuits on the level of total SCFAs. NC vs. MC: the different lowercase letters at the top of graph bars represent statistical significance (*p* < 0.05). MC vs. PC, LD, MD, and HD: the different lowercase letters at the top of graph bars represent statistical significance (*p* < 0.05).

SCFAs, including acetic, propionic, and butyric acid, serve as crucial intermediaries connecting nutritional intake, gut microbiota composition, and biological processes in health and disease. To investigate the microbiota-mediated regulatory effects of dietary fiber from biscuits, we conducted a quantitative analysis of straight-chain SCFA production in the murine cecal contents following five different fiber fermentations. Experimental data revealed a significant elevation (*p* < 0.05) in total SCFA levels in the PC, MD, and HD groups compared to the MC group, with the LD group exhibiting an increased acetic acid concentration (Figure 8). The MD group had notable increases in both acetic acid and propionic acid, while the HD group demonstrated elevated propionic and valeric acid levels. These findings suggest that the consumption of biscuits modulated SCFA production and enhanced the intestinal concentration of acetic, propionic, valeric, butyric, and caproic acids in hyperlipidemic mice. This regulatory mechanism may support gastrointestinal well-being and have a beneficial impact on metabolic health in humans.

### 3.9. Effects of Biscuits on Intestinal Flora in Mice

**Figure 9:**
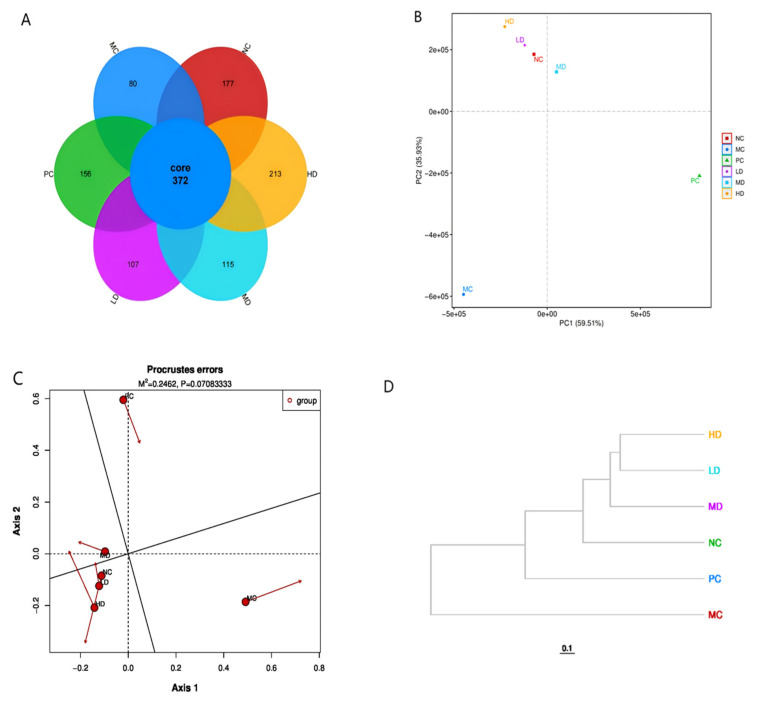
Effects of biscuits on the intestinal flora of mice. (**A**) Venn diagram, (**B**) PCA analysis results, (**C**) Procrustes analysis results, and (**D**) dendrogram of the distance between samples.

Figure 9A shows that 372 operational taxonomic units (OTUs) are shared across the experimental groups. The NC, MC, PC, LD, MD, and HD groups exhibited distinct OTU counts of 177, 80, 156, 107, 115, and 213, respectively. Comparative analysis revealed that the HD group maintained the largest OTU quantity, followed by the NC and PC groups. The MC group exhibited the smallest count. Notably, OTU numbers progressively increased across the LD, MD, and HD groups, corresponding with the intake of biscuits. The HD group values surpassed those of the NC and PC groups.

In PCA, the closer two samples are in the figure, the more similar their composition. The horizontal and ordinate axes in the PCA plots explained 59.51% and 35.93% of the interpretation degree for the overall difference between the samples, respectively. The MC group formed a separate cluster from the NC group in the PCA space, indicating marked structural differences in the microbial community. The intervention groups (HD, MD, and LD) receiving gastric-administered biscuits demonstrated progressive migration toward the NC group in ordination space (Figure 9B). This spatial pattern suggests that the biscuits effectively ameliorated high-fat diet-induced gut microbiota dysbiosis, outperforming the positive PC group in microbial community restoration.

Elevated similarity between samples in the Procrustes analysis corresponds to the tighter clusters observed in (Figure 9C), with directional markers illustrating discrepancies between species abundance and functional composition. The results from this analysis reveal that the MC group exhibited a clear separation from the NC cohort, highlighting distinct microbial community profiles between these experimental groups. Notably, the LD, MD, and HD groups clustered more closely with the NC group distribution pattern. This spatial relationship suggests that dietary supplementation with biscuits contributed to the restoration of gut microbiota balance disrupted by the high-fat diet, demonstrating superior efficacy compared to the PC group.

A dendrogram of the distances between samples serves as a tool for assessing microbial community resemblance across sample groups. As illustrated in (Figure 9D), branch length within the sample distance dendrogram directly reflects the genetic divergence levels. Closely clustered branches demonstrate higher taxonomic overlap, with proximal node positions signifying greater compositional congruence in microbial populations. Analysis of the sample clustering patterns revealed that the LD, MD, and HD experimental groups exhibited the strongest phylogenetic alignment with the NC group. This phylogenetic proximity suggests that biscuit intervention effectively mitigated high-fat diet-induced dysbiosis, resulting in an intestinal community composition similar to that of the NC group.

**Figure 10:**
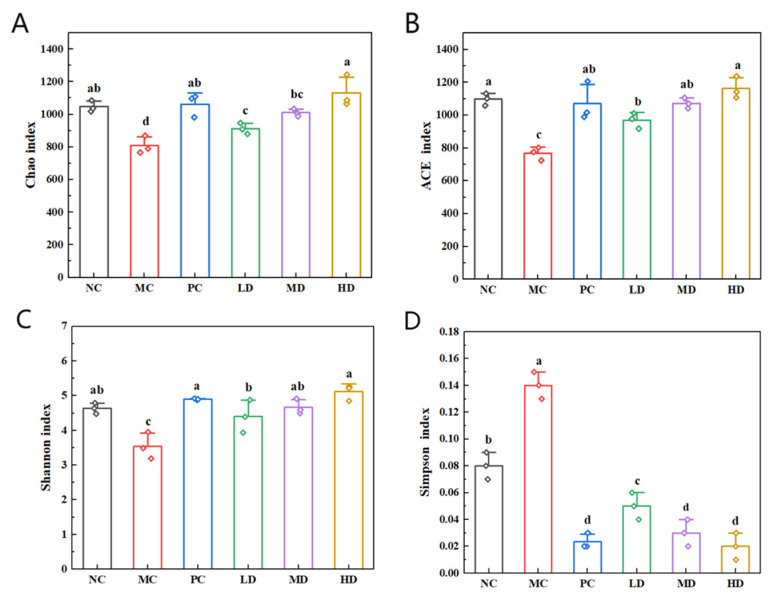
Alpha diversity assessment of biscuits on the intestinal flora of mice. (**A**) Chao index, (**B**) ACE index, (**C**) Shannon index, and (**D**) Simpson index. NC vs. MC: the different lowercase letters at the top of graph bars represent statistical significance (*p* < 0.05). MC vs. PC, LD, MD, and HD: the different lowercase letters at the top of graph bars represent statistical significance (*p* < 0.05).

As shows in Figure 10, the Chao and ACE indices were employed to measure species richness, while the Shannon and Simpson indices were used to evaluate community diversity. Elevated Shannon values and reduced Simpson values indicate greater biodiversity within communities. Analysis of the two diversity indices illustrated that the MC group exhibited markedly reduced intestinal microbiota diversity compared to the other groups (*p* < 0.05). This demonstrates that prolonged high-fat dietary intake substantially diminished microbial diversity in the murine intestinal ecosystem. Following intervention with biscuits, a significant increase in the Shannon index (*p* < 0.05) and a decrease in the Simpson index (*p* < 0.05) were observed. The results reveal that both the biscuit supplementation and lovastatin administration effectively enhanced microbial diversity, with the HD group exhibiting effects comparable to those of the PC group (*p* > 0.05). In terms of species richness metrics, the higher the Chao and ACE indices, the higher the species richness. The MC group had significantly lower Chao and ACE indices than the other experimental groups (*p* < 0.05), confirming that chronic high-fat consumption reduces microbial richness. Post-intervention data demonstrated substantial elevation of the Chao and ACE indices in the treatment groups, with the HD group outperforming the PC group (*p* < 0.05). This indicates the superior efficacy of the biscuit intervention in restoring microbial abundance. These findings collectively demonstrate that biscuits effectively ameliorate structural damage to the intestinal microbiota induced by the high-fat diet. Additionally, such intervention significantly enhances both diversity and richness in obese mice while addressing microbial community balance.

**Figure 11:**
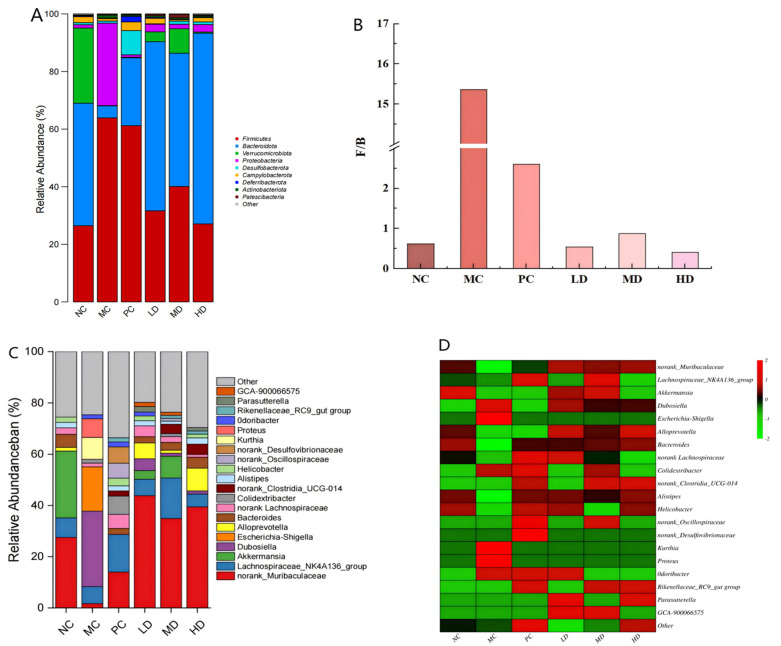
The regulatory effects of biscuits on the intestinal microbial community of mice with hyperlipidemia. (**A**) Species composition at the phyla level, (**B**) F/B ratio, (**C**) species composition at the genus level, and (**D**) species abundance heatmap at the genus level.

At the phylum classification level, the dominant bacterial groups in the intestinal microbiota across the different groups primarily included Firmicutes, Bacteroidota, Actinobacteria, Proteobacteria, and Verrucomicrobia. Comparative analysis revealed substantial alterations in gut microbiota composition between the MC group and the NC group. The MC group exhibited a marked reduction in Bacteroidota and Verrucomicrobia, contrasted by significant proliferation of Proteobacteria and Firmicutes (Figure 11A). Notably, Proteobacteria serve as a biomarker of microbial imbalance. Although harmless at normal levels, their overgrowth correlates with gastrointestinal inflammation and dysbiosis [34]. Intervention with lovastatin or biscuits effectively suppressed Proteobacteria proliferation and specifically enhanced Verrucomicrobia abundance in the LD and MD treatment groups. This demonstrates the therapeutic potential of these dietary interventions in restoring high-fat diet-induced microbiota balance.

The high-fat diet notably altered the Firmicutes/Bacteroidota (F/B) ratio, a recognized indicator of intestinal homeostasis. The MC group exhibited elevated Firmicute levels coupled with diminished Bacteroidota populations, resulting in an increased F/B ratio (Figure 11B). Clinical studies indicate that elevated F/B values are associated with metabolic dysregulation, thereby linking with obesity, non-alcoholic fatty liver disease (NAFLD), and cardiovascular risk [35]. This ratio elevation enhances microbial energy harvesting, promoting lipid/cholesterol biosynthesis and contributing to the development of metabolic syndrome [36]. The therapeutic administration of biscuits significantly reduced F/B values in the LD, MD, and HD groups (*p* < 0.05), effectively modulating the Firmicutes–Bacteroidota equilibrium. The biscuit intervention decreased the proportion of Firmicutes and Proteobacteria while restoring Bacteroidota and Verrucomicrobia, thereby partially restoring the ecological balance of the gut to a certain extent.

The 20 most abundant microbial taxa within the murine intestinal microbiota were identified via genus-level taxonomic profiling for further detailed examination. The dominant genera were norank_Muribaculaceae, Lachnospiraceae_NK4A136_group, Akkermansia, Dubosiella, Alloprevotella, Bacteroides, norank_Lachnospiraceae, Colidextribacter, norank_Clostridia_UCG-014, Alistipes, and unclassified_Lachnospiraceae, along with other numerically significant taxa. This hierarchical analysis revealed distinct microbial community patterns through quantitative assessment of the relative abundance distribution across experimental groups (Figure 11C).

As illustrated in Figure 11C, notable variations in microbial abundance were revealed across experimental groups. The NC group microbiota predominantly featured norank_Muribaculaceae (27.55%), Akkermansia (26.11%), Lachnospiraceae_NK4A136_group (6.66%), and Bacteroides (5.13%). The MC group microbiota demonstrated distinct characteristics, with Dubosiella (29.43%), Escherichia-Shigella (17.34%), Kurthia (8.54%), Proteus (7.28%), and Lachnospiraceae_NK4A136_group (6.66%) as the primary constituents.

In contrast, the PC group exhibited a diversified profile, comprising norank_Muribaculaceae (14.01%), Lachnospiraceae_NK4A136_group (14.65%), Colidextribacter (7.16%), norank_Desulfovibrionaceae (6.40%), norank_Oscillospiraceae (5.83%), and norank_Lachnospiraceae (5.48%). The LD group’s microbial configuration included norank_Muribaculaceae (43.84%), Lachnospiraceae_NK4A136_group (6.38%), Alloprevotella (6.17%), Dubosiella (4.64%), norank_Lachnospiraceae (4.16%), and Akkermansia (3.40%).

Notable shifts occurred in the MD group, which comprised norank_Muribaculaceae (34.98%), Lachnospiraceae_NK4A136_group (15.75%), Akkermansia (8.47%), norank_Clostridia_UCG-014 (3.66%), and Bacteroides (3.02%). The HD group results included norank_Muribaculaceae (39.47%), Alloprevotella (8.86%), Lachnospiraceae_NK4A136_group (4.96%), Bacteroides (4.27%), and norank_Clostridia_UCG-014 (4.11%). Correlation analysis revealed that the post-intervention microbial composition of the LD, MD, and HD groups exhibited similarities with the NC group.

As illustrated in (Figure 11D), compared with the NC group, the MC group exhibited a marked reduction in the relative abundance of norank_Muribaculaceae, Akkermansia, norank_Lachnospiraceae, Lachnospiraceae_NK4A136_group, Alloprevotella, Bacteroides, and Alistipes. Conversely, a substantial increase was observed in Dubosiella, Escherichia-Shigella, Kurthia, Proteus, Odoribacter, and Colidextribacter. These findings suggest that the high-fat diet contributed to the depletion of advantageous gut microbiota while promoting the proliferation of detrimental microbial species, thereby modifying intestinal flora composition and influencing lipid metabolic processes.

Compared to the MC group, the PC group exhibited a notable elevation in norank_Muribaculacea, norank_Lachnospiraceae, Lachnospiraceae_NK4A136_group, norank_Clostridia_UCG-014, Rikenellaceae_RC9_gut_group, and norank_Oscillospiraceae. This was accompanied by diminished levels of Kurthia and Proteus. No significant alterations were detected in Akkermansia, Alloprevotella, Dubosiella, or Escherichia-Shigella. However, increases in potentially harmful bacterial strains, including norank_Desulfovibrionaceae, Colidextribacter, and Helicobacter, were observed.

Following the administration of biscuits, the LD, MD, and HD groups exhibited a marked elevation in the relative abundance of norank_Muribaculacea, Alloprevotella, Bacteroides, and Alistipes genera, with notable reductions in Dubosiella, Escherichia-Shigella, Colidextribacter, Kurthia, and Proteus. The LD and MD groups demonstrated substantial enrichment of advantageous microorganisms, including GCA-900066575, Akkermansia, and norank_Lachnospiraceae, with Parasutterella (a unique beneficial bacterial strain absent in other experimental groups) being present in these groups. The Lachnospiraceae_NK4A136_group was only found exclusively in the MD group, while both the MD and HD groups exhibited a shared increased abundance of norank_Clostridia_UCG-014 and Rikenellaceae_RC9_gut_group, the latter being recognized for its short-chain fatty acid biosynthesis capabilities. These findings underscore dietary intervention as a principal modulator of gut microbial diversity, specifically highlighting the pronounced effects of biscuits and a high-fat diet on intestinal flora composition.

The experimental results demonstrate that consumption of biscuits effectively counterbalanced high-fat diet-induced microbial dysbiosis, significantly enhancing beneficial taxa such as norank_Muribaculaceae, Akkermansia, Alloprevotella, Bacteroides, norank_Lachnospiraceae, and Alistipes while suppressing pathogenic organisms including Dubosiella, Escherichia-Shigella, Kurthia, Proteus, and Colidextribacter. This intervention notably promoted specialized microbial consortia such as Parasutterella, norank_Clostridia_UCG-014, Rikenellaceae_RC9_gut_ group, and GCA-900066575. These changes led to the reconstitution of the gut microbial equilibrium, the optimization of lipid metabolic pathways, and the preservation of intestinal barrier integrity.

### 3.10. Correlation Between Biochemical Indices Related to Hyperlipidemic Intestinal Microflora and Short-Chain Fatty Acids

**Figure 12:**
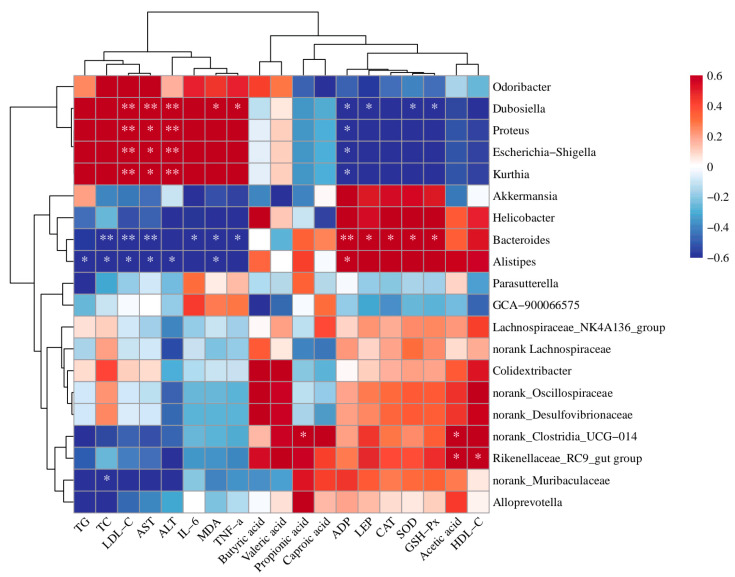
Correlation analysis between biochemical indices related to hyperlipidemic intestinal microflora and short-chain fatty acids. *p* < 0.05, *; *p* < 0.01, **.

To elucidate the relationship between intestinal microbiota alterations induced by biscuits and hyperlipidemia, we employed Spearman’s correlation method to examine associations among gut microorganisms, short-chain fatty acids, and multiple hyperlipidemia-related biomarkers. The analysis focused on the 20 most abundant genera; the results are shown in Figure 12. The results show that acetic acid demonstrated a significant positive correlation with norank_Clostridia_UCG-014 and Rikenellaceae_RC9_gut_group (*p* < 0.05), and propionic acid was significantly positively correlated with the relative abundance of norank_Clostridia_UCG-014 (*p* < 0.05). Notable microbial interactions included a positive correlation between Rikenellaceae_RC9_gut_group and HDL-C levels (*p* < 0.05) and a significant association between Alistipes abundance and ADP concentration (*p* < 0.05). Bacteroides populations exhibited multiple positive relationships, showing a significant correlation with LEP, CAT, SOD, and GSH-Px (*p* < 0.05), along with an elevated significant association with ADP (*p* < 0.01). Dubosiella demonstrated a significant positive correlation with MDA and TNF-α (*p* < 0.05), along with a notable association with LDL-C, AST, and ALT levels (*p* < 0.01). The genera Escherichia-Shigella, Kurthia, and Proteus had a significant positive relationship with AST (*p* < 0.05) and a highly significant correlation with ALT and LDL-C (*p* < 0.01). The results of this study suggest that ingestion of biscuits may alleviate lipid metabolism disorders, improve liver function indicators, reduce oxidative damage, enhance antioxidant capacity, significantly reduce serum pro-inflammatory factors, increase the secretion of leptin and adiponectin, and alleviate the occurrence of hyperlipidemia in mice. This may be through alterations in the intestinal flora and the generation of short-chain fatty acids.

## 4. Discussion

Hyperlipidemia is a significant worldwide health concern that not only disrupts the metabolic equilibrium but also elevates the risk of diseases endangering human health [37]. Current pharmacological options have limited effectiveness and can cause significant adverse reactions. Therefore, there is an urgent need to identify safe and economical lipid-lowering interventions. Functional food products have emerged as promising therapeutic alternatives due to their natural composition and favorable safety profiles. Nutritional approaches centered on whole grains are gaining validation, with research indicating their ability to manage lipid disorders and obesity [38]. Biscuits incorporate multiple bioactive components, including dietary fiber, β-glucan, and flavonoid compounds like rutin, anthocyanin, and saponins. The potential hypolipidemic effects observed in murine models likely stem from the combined action of these nutrients, particularly through the synergistic interaction of dietary fibers and β-glucan with mineral cofactors. Nevertheless, further investigation is required to determine the precise biochemical pathways involved in their therapeutic mechanisms.

Following a four-week intervention with oral administration of varying dosages of biscuits, notable physiological changes were observed. The MC group exhibited a marked increase in body mass, with histological examination revealing substantial lipid depositions in hepatic tissues. Conversely, the LD, MD, and HD groups demonstrated amelioration of hepatic steatosis, as evidenced by decreased serum AST and ALT levels, as well as reduced TC and TG concentrations. As a primary metabolic organ, hepatic tissue is crucial to the regulation of metabolic processes and lipid homeostasis. Previous investigations have documented the lipid-lowering potential of whole-grain dietary fibers in lipidemic mouse models [39]. Excessive lipid accumulation in hepatic tissues may induce oxidative stress and inflammatory responses, potentially leading to hepatic dysfunction [40]. Concordant findings from histomorphological assessments of liver and epididymal adipose tissue have corroborated these observations, substantiating the therapeutic efficacy of biscuits in mitigating lipid deposition and alleviating pathological alterations in a murine model of hyperlipidemia.

Research has indicated that hyperlipidemia primarily arises from oxidative stress and inflammatory processes [41]. The pathological progression of this condition involves oxidative mechanisms connecting lipid metabolism impairment to subsequent inflammatory reactions and apoptosis. Antioxidant enzymes, including glutathione peroxidase, catalase, and superoxide dismutase, serve crucial functions in mitigating hepatic damage and oxidative stress induced by lipid-rich diets [31]. Malondialdehyde concentration, which reflects lipid peroxidation intensity, serves as a reliable biomarker for hepatic oxidative damage [15]. Concurrently, elevated levels of the pro-inflammatory cytokines IL-6 and TNF-α have been documented in hyperlipidemic murine models [42]. Our experimental data demonstrated that dietary intervention with biscuits notably enhanced antioxidant enzyme activity (SOD, CAT, and GSH-Px) and reduced MDA levels in hyperlipidemic mice. This treatment concurrently decreased serum concentrations of TNF-α and IL-6, indicating substantial anti-inflammatory effects. These findings align with previous reports documenting that dietary fiber can modulate inflammatory mediators, enhance antioxidant defenses, and reduce oxidative stress markers [43].

Contrary to the elevated leptin concentration typically observed in obese individuals, the MC group exhibited reduced leptin levels compared to the NC group [44]. Contemporary classifications of obesity distinguish between hypoleptinemia, characterized by inadequate hormone production, and hyperlipidemia, which is associated with leptin resistance. Our observation of diminished leptin levels parallels rare cases of human obesity resembling the pathophysiology of type I diabetes [45]. The existing literature has documented the impaired secretion of leptin and adiponectin in NAFLD and hyperlipidemia models. Our study confirms these findings, showing that biscuits effectively restore LEP and ADP secretion in hyperlipidemic mice, thereby corroborating previous research outcomes.

The therapeutic benefits of biscuits in mitigating high-fat diet-induced hyperlipidemia appear to be linked to their capacity to elevate SCFAs and restore gut microbial balance. These SCFAs are synthesized by commensal microorganisms that metabolize complex carbohydrates and resistant fibers, generating metabolites crucial to gastrointestinal integrity. SCFAs exhibit multifaceted biological activities, including microbial community modulation, immune enhancement, and lipid metabolism regulation. Butyric acid has been shown to suppress NF-κB signaling pathway activation in hepatic macrophages, thereby mitigating the inflammatory response. Acetic acid has been shown to stimulate mitochondrial biogenesis and AMPK activation, promoting hepatic lipid oxidation [46,47]. In addition, propionic acid increases leptin production two-fold, effectively suppressing lipogenesis [48]. It has been reported that improvements in gut microbiota composition can increase the production of short-chain fatty acids [49]. We quantitatively analyzed the direct chain SCFAs produced by the fermentation of five dietary fiber sources in the cecal contents of mice. The study revealed that biscuits modulated microbial SCFA production by notably increasing intestinal concentration of acetic, propionic, butyric, valeric, and caproic acids in hyperlipidemic mice, thereby supporting intestinal homeostasis. These findings align with the existing scientific literature [50].

Research has demonstrated that intestinal microbial populations exert a significant influence on lipid metabolism regulation [51]. These microorganisms influence both digestive processes and hepatic functions through the gut–liver communication pathway. Dietary patterns that are rich in lipids can induce structural alterations in intestinal flora composition, potentially disrupting metabolic homeostasis and lipid equilibrium [52]. Extensive research has demonstrated that microbial dysbiosis in patients with metabolic disorders such as dyslipidemia, diabetes mellitus, and hepatic steatosis is characterized by elevated levels of pro-inflammatory pathogens and a diminished presence of beneficial bacterial strains [53]. Emerging scientific evidence supports microbiota modulation as a viable strategy for managing lipid abnormalities [54]. These findings highlight the pivotal involvement of microbiota in metabolic disorder pathogenesis. Current investigations have revealed that dietary interventions utilizing functional nutrients can stimulate beneficial microbial metabolite production, suggesting their therapeutic potential in lipid regulation [54,55]. Substantial evidence has shown the involvement of microbial-derived metabolites in the pathology of hyperlipidemia [55]. Consequently, targeted modulation of the intestinal flora and associated lipid metabolic through nutritional intervention presents a promising approach for lipid management [56].

The findings of previous studies have demonstrated that individuals with hyperlipidemia exhibit a marked reduction in Bacteroidetes and Actinobacteria and elevated levels of Proteobacteria and Firmicutes relative to healthy individuals. The proportion of advantageous microbial species, such as norank_Muribaculaceae, Akkermansia, Alloprevotella, Bacteroides, norank Lachnospiraceae, and Alistipes, appears particularly diminished. In addition, diets rich in fats have been found to be correlated with diminished microbial diversity in the gut, proliferation of Gram-negative organisms like Enterobacteriaceae, the depletion of advantageous microbes including Bifidobacterium, and reductions in the Actinobacteria phylum, which has anti-inflammatory properties. Intestinal microbial communities influence hepatic lipid metabolism through modulation of dietary calorie utilization, which is a key role of the gut microbiota in hyperlipidemia. Bacteroidetes and Firmicutes represent over 90% of the murine gut microbiota and exhibit distinct efficiency in harvesting dietary energy [57]. A diminished Firmicutes/Bacteroidetes ratio is strongly associated with reduced caloric yield from microbial dietary processing [58]. Nutritional intervention utilizing biscuits demonstrated preventive effects in hyperlipidemic mice through F/B ratio modulation, thereby limiting dietary energy absorption.

This study reveals that intragastric administration of biscuits to hyperlipidemic mice resulted in decreased abundance of Firmicutes and Proteobacteria while enhancing populations of beneficial microbiota, including norank_Muribaculaceae, Akkermansia, Alloprevotella, Bacteroides, and Rikenellaceae_RC9_gut_group. Alloprevotella, which is recognized for its anti-inflammatory properties, serves as a beneficial microbial species. Research has indicated that Bacteroides and Akkermansia help modulate or ameliorate the inflammatory response through dietary fiber fermentation, producing essential metabolites that support intestinal homeostasis, such as SCFAs [58,59]. At the genus level, Akkermansia and Bifidobacterium exhibit consistent protective effects against hyperlipidemia development and progression [60]. Current findings align with previous reports that the lipid-lowering efficacy of whole-grain oat flavonoids is correlated with elevated Akkermansia, Bifidobacterium, and norank_f_Muribaculaceae [61]. The observed elevation in Akkermansiaceae and norank_f_Muribaculaceae corresponded with improved hyperlipidemic parameters in mice fed a high-fat diet. Concurrent reduction in potentially harmful genera, including Escherichia-Shigella, Dubosiella, and Colidextribacter, is consistent with previous reports [62,63]. This research further validates the therapeutic potential of biscuits in restoring gut microbiota balance disrupted by a lipid-rich diet.

Individuals tend to be more receptive to dietary interventions that can be seamlessly integrated into their daily routines, as opposed to pharmacological therapies. Consequently, dietary interventions centered on functional foods have demonstrated remarkable efficacy in managing and preventing hyperlipidemia, garnering widespread recognition within the global scientific community. Notably, dietary patterns exert a profound influence on gut microbial composition. Whole grains serve as a vital nutritional substrate for intestinal flora, promoting microbial diversity and ecological balance. These microbial communities enzymatically break down whole-grain components into bioactive metabolites while simultaneously facilitating host–nutrient interplay [64]. Our experimental findings corroborate this mechanism. Spearman algorithm analysis showed that the biscuits effectively ameliorated hyperlipidemia via gut microbiota modulation. This makes intervention with sugar-free biscuits, which contain multiple synergistic grains, particularly suitable as a long-term dietary strategy.

This investigation demonstrated that biscuits effectively reduced blood lipid levels through multiple mechanisms, including lipid profile optimization, oxidative stress mitigation, inflammatory response suppression, and modulation of SCFAs and gut microbiota composition. These collective findings demonstrate the significant potential of these biscuits in hyperlipidemia prevention. Experimental data suggest that these biscuits can serve as a promising strategy for relieving lipid metabolism disorders. These products could, in the future, be formulated into specialized medical foods or used as a nutraceutical supplement for regular dietary integration.

Although this study offers a novel perspective on nutritional interventions for lipid regulation, it has certain limitations that warrant consideration. Subsequent investigations should prioritize examining the synergistic and antagonistic interactions among the bioactive compounds in whole-grain formulations, as dosage-dependent efficacy variations may critically influence therapeutic outcomes. Concurrently, translational research must address practical challenges, including establishing equivalent dosage conversion and determining appropriate intake recommendations for human application. Comprehensive mechanistic studies are an essential prerequisite prior to clinical implementation.

## 5. Conclusions

Experimental data reveal that biscuits demonstrate efficacy in alleviating hyperlipidemia in mice through multiple mechanisms. The biscuits notably decreased body mass and fasting glucose levels while enhancing hepatic functional parameters. The intervention also attenuated oxidative stress markers and boosted antioxidant enzyme activity, concurrently lowering serum concentrations of pro-inflammatory cytokines. Significant elevations in leptin and adiponectin secretion were observed, along with mitigation of histopathological alterations in hepatic and epididymal adipose tissues.

The biscuits modulated gut microbial ecology by elevating the intestinal concentration of SCFAs, including acetic, propionic, butyric, caproic, and valeric acids. Microbial community analysis revealed regulatory effects on phylum-level distribution (Firmicutes, Bacteroidetes, Actinobacteria, and Proteobacteria), promoting beneficial genera (norank_Muribaculaceae, Akkermansia, Alloprevotella, Bacteroides, norank_Lachnospiraceae, and Alistipes) while suppressing potentially pathogenic taxa (Dubosiella, Escherichia-Shigella, Kurthia, Proteus, and Colidextribacter). This microbial remodeling contributed to lipid metabolism regulation, intestinal barrier maintenance, and dysbiosis correction.

These findings substantiate the potential of biscuits in hyperlipidemia management through multi-target mechanisms such as the metabolic regulation of lipids, anti-inflammatory effects, and microbiome modulation. This innovative investigation into the lipid-lowering mechanisms of these whole-grain products establishes a scientific foundation for their application in chronic disease prevention and dietary intervention. Highland barley tartary buckwheat coarse-grain biscuits have proven to be a promising functional food alternative for hyperlipidemia management, addressing a current research gap in the improved application of whole grains.

## Figures and Tables

**Table 1:** Organ index and fat index in each group.

Group	Cardiac Index	Liver Index	Spleen Index	Pulmonary Index	Renal Index	Epididymal Adipose Index
NC	5.94 ± 0.31 ^a^	30.20 ± 1.37 ^e^	2.60 ± 0.36 ^a^	6.19 ± 0.20 ^ab^	12.97 ± 0.39 ^ab^	11.87 ± 0.39 ^e^
MC	5.85 ± 0.12 ^ab^	40.30 ± 2.26 ^a^	2.40 ± 0.18 ^ab^	6.35 ± 0.27 ^a^	13.43 ± 0.33 ^a^	23.76 ± 1.11 ^a^
PC	5.45 ± 0.23 ^c^	33.13 ± 1.23 ^cd^	2.37 ± 0.16 ^ab^	6.21 ± 0.17 ^ab^	13.10 ± 0.37 ^ab^	14.57 ± 0.61 ^d^
LD	5.64 ± 0.26 ^bc^	35.09 ± 1.28 ^b^	2.39 ± 0.14 ^ab^	6.41 ± 0.23 ^a^	13.08 ± 0.61 ^ab^	20.15 ± 0.78 ^b^
MD	5.84 ± 0.32 ^ab^	34.59 ± 1.36 ^bc^	2.39 ± 0.13 ^ab^	6.44 ± 0.19 ^a^	12.91 ± 0.16 ^ab^	18.55 ± 0.32 ^c^
HD	5.64 ± 0.38 ^bc^	32.20 ± 1.31 ^d^	2.38 ± 0.10 ^ab^	6.47 ± 0.18 ^a^	12.89 ± 0.12 ^b^	14.13 ± 1.26 ^d^

Note: NC vs. MC: the means marked with different superscript lowercase letters represent statistical significance (*p* < 0.05). MC vs. PC, LD, MD, and HD: the means marked with different superscript lowercase letters represent statistical significance (*p* < 0.05).

## Data Availability

The original contributions presented in the study are included in the article, and further inquiries can be directed to the corresponding author.

## Abbreviations

The following abbreviations are used in this manuscript:

FBS	fasting blood-glucose
TG	triglyceride
TC	total cholesterol
LDL-C	low-density lipoprotein cholesterol
HDL-C	high density lipoprotein cholesterol
ALT	alanine aminotransferase
AST	aspartate aminotransferase
GSH-Px	glutathione peroxidase
MDA	malondialdehyde
SOD	superoxide dismutase
CAT	catalase
IL-6	interleukin 6
TNF-α	tumor necrosis factor alpha
LEP	leptin
ADP	adiponectin
SCFAs	short-chain fatty acids
